# Research on decoupling control for the longitudinal and lateral dynamics of a tractor considering steering delay

**DOI:** 10.1038/s41598-022-18335-0

**Published:** 2022-08-17

**Authors:** Hequan Miao, Peisong Diao, Guangfei Xu, Wenyan Yao, Zhicai Song, Wenjun Wang

**Affiliations:** 1 College of Agricultural Engineering and Food Science, Shandong University of Technology, Zhangdian District, Zibo, 255000 Shandong China; 2 Liaocheng Academy of Agricultural Sciences, Liaocheng, China

**Keywords:** Electrical and electronic engineering, Mechanical engineering

## Abstract

To enhance the efficiency of tractor operation, the longitudinal and lateral dynamic control of a self-driving tractor is studied in this paper, and a control system that decouples control of the longitudinal and lateral movement of the tractor is proposed. The trajectory controller calculates the desired speed and desired yaw rate signals of each subsystem, and a PID controller regulates the longitudinal speed of the tractor. A pure pursuit algorithm calculates the desired front wheel angle of the tractor. To decrease the system time delay in the automatic steering system, an automatic steering scheme based on improved Smith predictive control is proposed. Through decoupling control of the longitudinal and lateral controllers, the tractor’s path tracking performance is assured.

## Introduction

The loss of young labour and the aggravation of the energy crisis have promoted the development of high efficiency and energy saving agricultural production. To enhance the efficiency of tractor operation, this paper studies the path tracking problem of autonomous driving tractors that many scholars have researched in this field. At present, methods commonly used for tractor path tracking are split into two categories: one is to control through mathematical models, and the other is to regulate methods that do not rely on mathematical models. According to the type of mathematical model used, they are divided into control methods based on a vehicle kinematics model and control methods based on a vehicle dynamics model^1^. According to the tracking direction, path tracking can be split into lateral tracking and longitudinal tracking. At present, the most used lateral tracking algorithms are PID, fuzzy control, optimal control and preview point control.

In terms of lateral tracking, some researchers have conducted correlated studies. Netto et al.^2^ used a PID control method for trajectory tracking, which has better tracking performance in lateral trajectory tracking, but has poor adaptability to complex situations. Kang et al.^3^ used a PID method to obtain steering input values. The model uses two PID controllers to control the lateral deviation and the heading deviation and calculates the steering input value through the output of the deviation controller. Luo et al.^4^ used a PID algorithm to design a path tracking controller for Dongfanghong X-804 tractor straight tracking, which uses the front wheel angle as the input and the lateral tracking deviation is the output. Bodur et al.^5^ proposed a double look-ahead reference point vehicle path tracking method, which uses only simple arithmetic operations. The adopted preview reference point can compensate for the centrifugal force and reduce the lateral deviation caused by the change in the path curvature. Zhang et al.^6^ used the fuzzy adaptive PID control method to control a transplanter’s steering operation. The deviation between the actual heading angle and the target heading angle was the difference from the actual front wheel angle, and the desired front wheel angle was calculated together with the driving speed. Plessen and Bemporad^7^ proposed a trajectory tracking method based on linear time-varying model predictive control theory for high-precision closed-loop offline path tracking in agricultural scenes. Wang and Hsu^8^ proposed an improved pure pursuit algorithm, which reduces lateral tracking deviation by regulating the heading angle and steering angle and improves the tracking accuracy compared with traditional methods. Li et al.^9^ proposed a path tracking method based on a fuzzy adaptive pure pursuit model, which improves the path tracking accuracy through adaptive adjustment of the forward-looking distance. Chen et al.^10^ designed an auxiliary driving system for a high-gap plant protection machine based on the preview algorithm and verified the driving stability and operation accuracy of the auxiliary driving system through simulation and real vehicle tests. A composite controller for wheeled mobile robots with external disturbances and parameter uncertainties was studied by Liu^11^. A disturbance observer based on an adaptive sliding mode dynamic controller was introduced to estimate disturbance online, adjust control gain automatically and eliminate the chattering phenomenon. Zhang et al.^12^ proposed a sliding mode method based on the integrated control algorithm of deviations of lateral position and heading angle. This strategy generates the corresponding steering angle by modifying the proportional coefficients of the lateral position controller and the heading angle controller. A comparison of the various lateral tracking control strategies is shown in Table 1.

**Table 1:** Comparison of lateral tracking control methods.

Control method	Dependent on mathematical model	Advantages	Disadvantages	Suitable system
PID method	No	Easy to implement	Poor adaptability of control parameters	Linear system
MPC method	Yes	It can solve the multiconstraint system, make up the uncertainty in time and enhance stability	Large amount of calculation	Nonlinear system
Pure pursuit	No	Less control parameters strong stability	Structure is fixed difficult to optimize	Nonlinear system
Sliding mode control	Yes	Strong anti-jaming ability	There is a chattering phenomenon	Nonlinear system
Fuzzy control	No	Strong robustness to parameter changes	Fuzzy rules are not systematic	Nonlinear system

Longitudinal tracking regulates the longitudinal speed or acceleration as the target. Because a tractor’s operating environment is relatively simple and the requirement for longitudinal motion tracking is relatively low, traditional PID control can satisfy the operation requirements. Researchers have conducted studies in the field of autonomous vehicle longitudinal motion control. Cao^13^ studied high-level unmanned vehicles, designed for speed tracking, and verified the input and output linearization of a sliding mode controller through a test for variable speed on a straight path to propose a sliding mode control strategy. Erdal^14^ studied the path tracking control of tractors, proposed a PD + T2FNN (Proportive-Derivative Controller + Typ-2 Fuzzy Neural Network) yaw-angle dynamics controller and PID controller for longitudinal speed control, and tested the effectiveness of the algorithm through actual vehicle tests.

Among the above methods, methods using kinematic and dynamic models largely depend on the accuracy of the mathematical model. In addition, a tractor is a complex nonlinear system in actual operation. It is difficult to establish a high-precision mathematical model, so the accuracy of the established model directly affects the efficiency of tractor path tracking. PID control and pure pursuit algorithms neither rely on complex mathematical models nor require complex control theory. They are widely used in the field of agricultural vehicle navigation control. At present, most researchers use the tractor steering angle as the control variable, and speed control research mainly focuses on the development of cruise control systems. There are few studies that combine longitudinal speed control and lateral motion control. The combined control of the longitudinal and lateral movement of a tractor can not only improve the efficiency of the tractor and reduce the labour intensity, but will also be more practical. Meanwhile, there is a lack of relevant research on steering time delay in the literature on steering subsystem research. When a tractor operates in a straight line in the field, it must maintain a steady speed to ensure the operation effect. When the head turns, the driving speed should be reduced during the turning process to ensure the smooth running of the tractor while achieving the smallest turning radius. To provide a technical reference for the realization of higher efficiency and higher precision unmanned driving, based on the inverse kinematics model, this study analyses the longitudinal and lateral control of a tractor and uses a pure pursuit algorithm to couple the longitudinal and lateral motion of the tractor. To improve the time delay of an automatic steering system, an improved Smith predictive control strategy is established, and the effectiveness of the improved control strategy is verified through simulation comparison.

This paper is organized as follows. The experimental plant is described in “Hardware construction of a path tracking control system” section. The modelling of the autonomous tractor is presented in “Model of an autonomous tractor” section. In “Research on the control strategy of an autonomous tractor” section, the overall control structure and the tractor control strategy are described. The simulation results of the whole system are given and analysed accordingly in “System simulation and testing” section. Finally, some conclusions are drawn from this study in “Conclusion” section.

## Hardware construction of a path tracking control system

**Figure 1:**
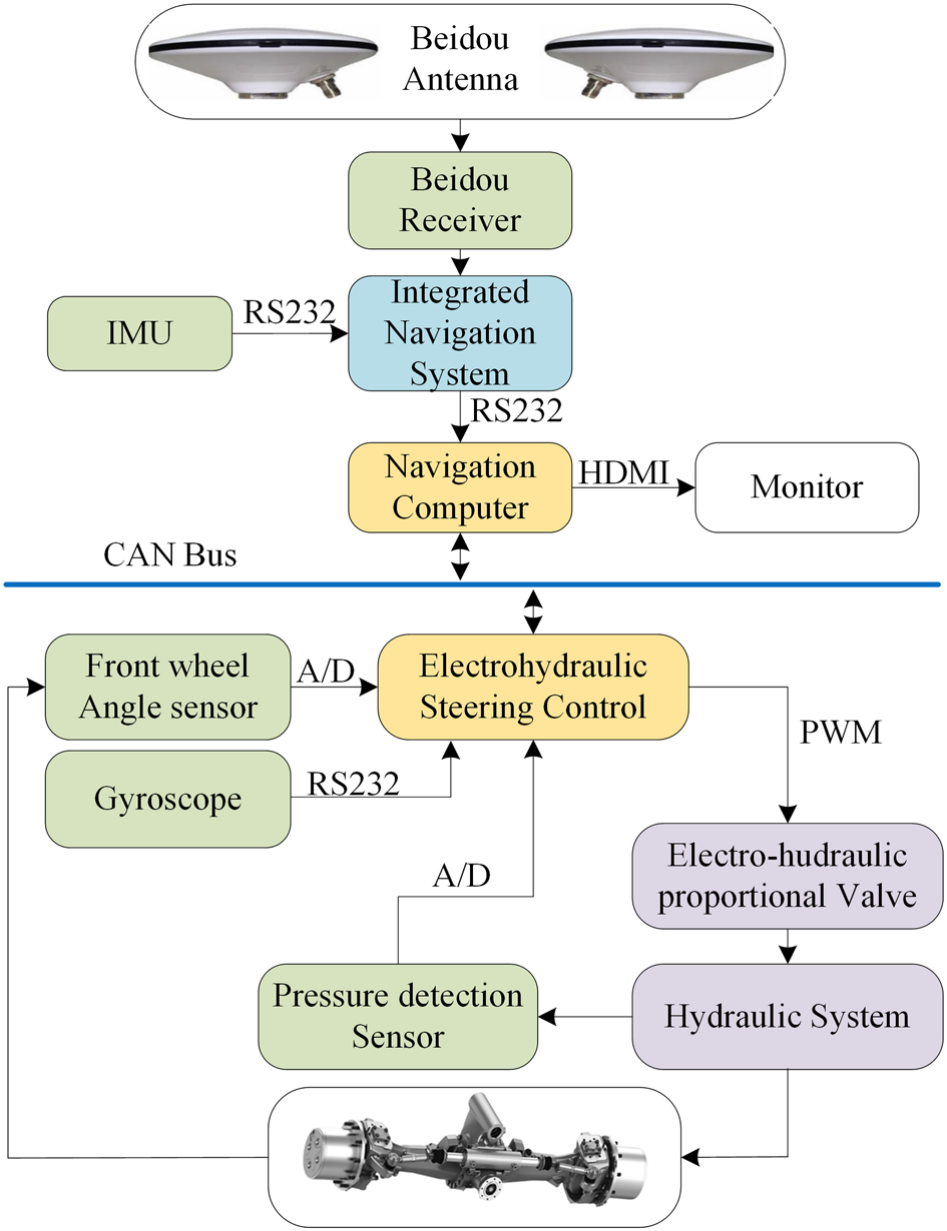
Hardware architecture of an autonomous tractor.

The hardware system structure of an automatic driving tractor is shown in Fig. 1. The experimental platform is composed of two subsystems, an automatic driving navigation system and an electronically controlled hydraulic steering system. The automatic driving navigation system is primarily composed of an integrated navigation system and a navigation computer. The integrated navigation system is composed of a double antenna positioning system and an inertial navigation system. A dual antenna positioning receiver is placed along the direction of the car body and is used to measure the vehicle’s heading angle. The inertial navigation system is used for the auxiliary satellite positioning system.

When the satellite signal is lost, the data calculated by the inertial navigation system can be used to locate the tractor through corresponding calculation. The inertial navigation system can also measure the tractor’s attitude angle information.

The navigation computer calculates the deviation value between the current trajectory and the reference trajectory based on the data transmitted by the integrated navigation system to provide the steering angle information for the automatic steering system to execute. The steering computer calculates the steering deviation value according to the steering angle instruction received and the real-time detection value obtained by the front wheel angle sensor and controls the hydraulic steering system to complete the steering operation. The pressure sensor in the figure is used to detect the oil pressure and prevent the operation of the automatic hydraulic steering system from overloading. The yaw rate and front wheel angle of the tractor are measured by a gyroscope and front wheel angle sensor, respectively.

## Model of an autonomous tractor

When a tractor is operating, it usually works with farm tools. Different farm tools need different traction forces during operation, and some farm tools need to be driven by the tractor’s power output to achieve the operation effect. Therefore, the tractor motion is simplified in this study, only the motion of the tractor itself is considered, and all the power of the tractor is used for its own driving.

### Kinematic model of a tractor

**Figure 2:**
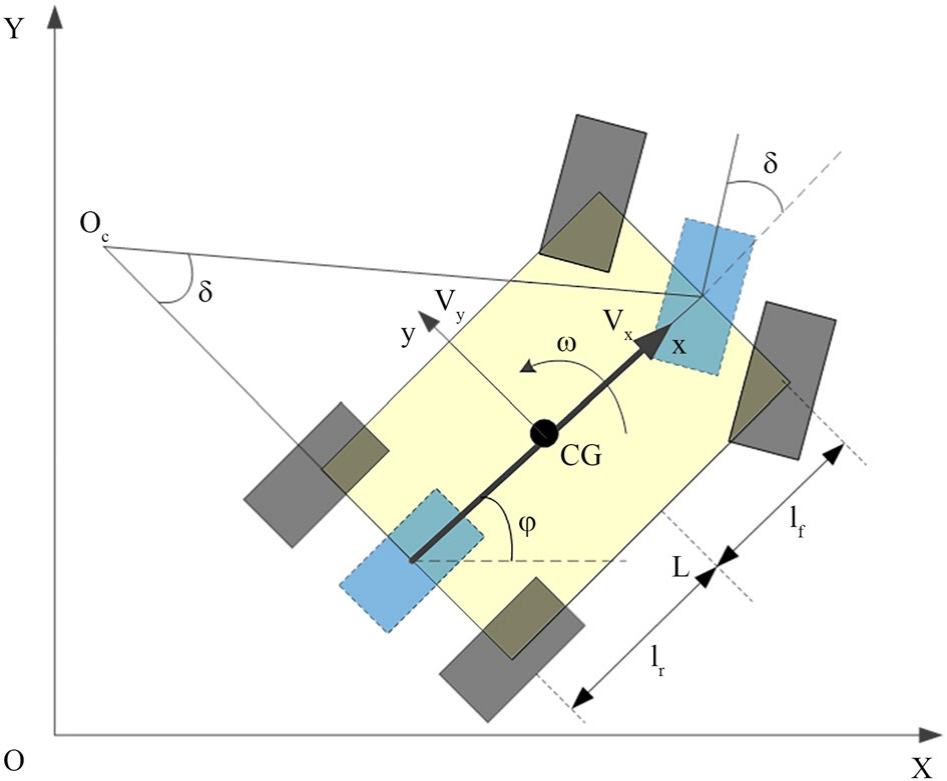
Two-degrees-of-freedom kinematics model (blue) at the CG.

Tractors are low-speed four-wheel vehicles. When considering errors such as tire and soil characteristics, tire slip, actuator transmission, installation and other errors, it is difficult to describe the system motion through an accurate mathematical model. Simplified mathematical models are usually used to describe the control system. This study uses the model shown in Fig. 2.

1 $$\left\{ {\begin{array}{*{20}l} {\dot{x} = V_{{\text{x}}} {\text{cos}} \; \varphi { - }V_{y} \sin \varphi } \hfill \\ {\dot{y} = V_{{\text{x}}} \sin \varphi + V_{y} \cos \varphi } \hfill \\ {\tan \delta = {{(\omega L)} \mathord{\left/ {\vphantom {{(\omega L)} {V_{{\text{x}}} }}} \right. \kern-\nulldelimiterspace} {V_{{\text{x}}} }}} \hfill \\ \end{array} } \right..$$

### Inverse kinematics model of a tractor

In the decoupling control of the longitudinal and lateral motion of a tractor, it is necessary to research the longitudinal speed and the yaw rate. The above tractor kinematic model can be further written as follows according to *V*_*y*_ = *ωl*_*r*_:

2 $$\left[ {\begin{array}{*{20}l} {\dot{x}} \\ {\dot{y}} \\ \end{array} } \right] = \left[ {\begin{array}{*{20}l} {\cos \varphi } & { - l_{r} \sin \varphi } \\ {\sin \varphi } & {l_{r} \cos \varphi } \\ \end{array} } \right]\left[ {\begin{array}{*{20}l} {V_{x} } \\ \omega \\ \end{array} } \right].$$

Because the coordinate of the CG is *P*_*CG*_ = [*x y*]^*T*^, the above formula can be expressed as:

3 $$\dot{P}_{CG} = \left[ {\begin{array}{*{20}l} {\dot{x}} \\ {\dot{y}} \\ \end{array} } \right] = \left[ {\begin{array}{*{20}l} {\cos \varphi } & { - l_{r} \sin \varphi } \\ {\sin \varphi } & {l_{r} \cos \varphi } \\ \end{array} } \right]\left[ {\begin{array}{*{20}l} {V_{x} } \\ \omega \\ \end{array} } \right].$$

The inverse kinematics model shown below is established.

4 $$\left[ {\begin{array}{*{20}l} {V_{x} } \\ \omega \\ \end{array} } \right] = \left[ {\begin{array}{*{20}l} {\cos \varphi } & {\sin \varphi } \\ {\frac{1}{{ - l_{r} }}\sin \varphi } & {\frac{1}{{l_{r} }}\cos \varphi } \\ \end{array} } \right]\left[ {\begin{array}{*{20}l} {\dot{x}} \\ {\dot{y}} \\ \end{array} } \right].$$

The desired longitudinal velocity and the desired yaw rate are expressed as:

5 $$\left[ {\begin{array}{*{20}l} {V_{xd} } \\ {\omega_{d} } \\ \end{array} } \right] = \left[ {\begin{array}{*{20}l} {\cos \varphi } & {\sin \varphi } \\ {\frac{1}{{ - l_{r} }}\sin \varphi } & {\frac{1}{{l_{r} }}\cos \varphi } \\ \end{array} } \right]\left[ {\begin{array}{*{20}l} {\dot{x}} \\ {\dot{y}} \\ \end{array} } \right].$$

In the above formula, *ω*_*d*_ is the desired yaw rate, and *V*_*xd*_ is the desired longitudinal speed. Martins et al.^15^ further expressed the above inverse kinematics formula as follows:

6 $$\left[ {\begin{array}{*{20}l} {V_{xd} } \\ {\omega_{d} } \\ \end{array} } \right] = \left[ {\begin{array}{*{20}l} {\cos \varphi } & {\sin \varphi } \\ {\frac{1}{{ - l_{r} }}\sin \varphi } & {\frac{1}{{l_{r} }}\cos \varphi } \\ \end{array} } \right]\left[ {\begin{array}{*{20}l} {\dot{x}_{d} + k_{s} \tanh (k_{e} e_{x} )} \\ {\dot{y}_{d} + k_{s} \tanh (k_{e} e_{y} )} \\ \end{array} } \right],$$

where *e*_*x*_ = *x*_*d*_ − *x* and *e*_*y*_ = *y*_*d*_ − *y* are the current position errors in the *X* and *Y* axes, respectively. *k*_*s*_ represents the saturation constant, *k*_*e*_ represents the gain of the controller,and both *k*_*s*_ and *k*_*e*_ > 0. The coordinates (*x, y*) and (*x*_*d*_*, y*_*d*_) are the current and the desired coordinates, respectively.

### Yaw motion of a tractor

**Figure 3:**
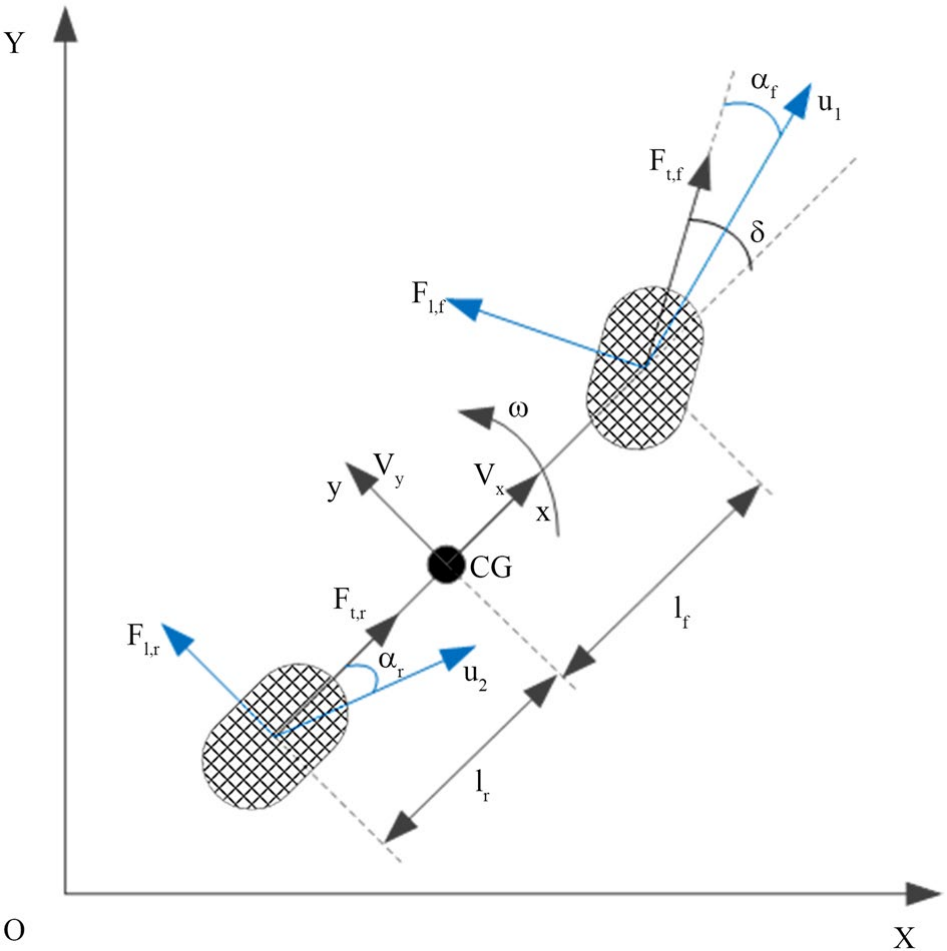
Tractor yaw dynamics model.

When a tractor actually operates, the driving speed is slow. It can be considered that the sideslip forces acting on the left and right wheels are equal. A three-degrees-of-freedom vehicle model is used to research the yaw, lateral and longitudinal motion of the tractor. The tractor’s speed, slip angle, and external force on the vehicle body are shown in Fig. 3. To facilitate the derivation of the tractor's yaw dynamics model, the following assumptions are made for the working conditions:

1. The air resistance of the tractor when driving is ignored.
2. Considering that the moments of the tires are small, they can be ignored.

The lateral movement of the tractor can be obtained according to the force balance:

7 $$F_{{{\text{tf}}}} \sin \delta + F_{lf} \cos \delta + F_{lr} = m(\dot{V}_{y} + \omega V_{x} ).$$

Here, $\dot{V}_{y} + \omega V_{x}$ represents the lateral component of the absolute acceleration of the CG. The law of rotation of a rigid body on a fixed axis is

8 $$M_{Z} = J\beta = J\dot{\omega },$$

where *J* represents the moment of inertia of a rigid body and *β* represents the angular acceleration of a rigid body. According to the definition of the moment of inertia

9 $$J = \sum\limits_{i = 1}^{n} {\Delta m_{i} r_{i}^{2} } .$$

The yaw motion of the tractor at the CG is written as Eq. (10) according to the moment balance.

10 $$(F_{{{\text{tf}}}} \sin \delta + F_{lf} \cos \delta ) - F_{lr} l_{r} = J\dot{\omega }.$$

To decrease the calculation strength of the tractor’s moment of inertia, Garrott^16^ approximated the moment of inertia as:

11 $$J = ml_{f} l_{r} .$$

### Tire model for tractor dynamics

Considering that a tire is a complex nonlinear system in actual operations, to facilitate the calculation of the tire lateral force, a linear model is used to simplify it. The lateral tire force has a linear relationship with the slip angle according to Gartley and Bevly^17^.

12 $$\left\{ {\begin{array}{*{20}l} {F_{lf} = k_{f} \alpha_{f} } \\ {F_{lr} = k_{r} \alpha_{r} } \\ \end{array} } \right.,$$

where *k*_*i*_, *i* = {*f,r*} represents the cornering stiffness of the tires.

The side-slip angle of the front and rear wheels is calculated with the following formula:

13 $$\left\{ {\begin{array}{*{20}l} {\alpha_{f} = \beta + \frac{{\omega l_{f} }}{{V_{x} }} - \delta } \\ {\alpha_{r} = \beta - \frac{{\omega l_{r} }}{{V_{x} }}} \\ \end{array} } \right.,$$

where

14 $$\beta = \frac{{V_{{\text{y}}} }}{{V_{x} }}.$$

Equation (13) can be written as:

15 $$\left\{ {\begin{array}{*{20}l} {\alpha_{f} = \frac{{V_{{\text{y}}} + \omega l_{f} }}{{V_{x} }} - \delta } \\ {\alpha_{{\text{r}}} = \frac{{V_{{\text{y}}} - \omega l_{{\text{r}}} }}{{V_{x} }}} \\ \end{array} } \right..$$

The equation of tractor yaw dynamics can be written in a state space form

16 $$\left[ {\begin{array}{*{20}l} {\dot{V}_{{\text{y}}} } \\ {\dot{\omega }} \\ \end{array} } \right] = \left[ {\begin{array}{*{20}l} {{\text{A}}_{11} } & {{\text{A}}_{{{12}}} } \\ {{\text{A}}_{21} } & {{\text{A}}_{22} } \\ \end{array} } \right]\left[ {\begin{array}{*{20}l} {V_{{\text{y}}} } \\ \omega \\ \end{array} } \right] + \left[ {\begin{array}{*{20}l} {B_{11} } \\ {B_{21} } \\ \end{array} } \right]\delta ,$$

where

$$\begin{array}{*{20}l} {{\text{A}}_{11} = - \frac{{k_{f} + k_{r} }}{{mV_{x} }}} & {{\text{A}}_{21} = \frac{{k_{r} l_{r} - k_{f} l_{f} }}{{I_{{\text{z}}} V_{x} }}} \\ {{\text{A}}_{12} = \frac{{k_{r} l_{r} - k_{f} l_{f} }}{{mV_{x} }} - V_{x} } & {{\text{A}}_{22} = - \frac{{k_{f} l_{f}^{2} + k_{r} l_{r}^{2} }}{{I_{{\text{z}}} V_{x} }}} \\ \end{array} ,$$

$$B_{11} = \frac{{k_{f} }}{m},B_{21} = \frac{{k_{f} l_{f} }}{{I_{z} }}.$$

### Longitudinal dynamics model of a tractor

**Figure 4:**
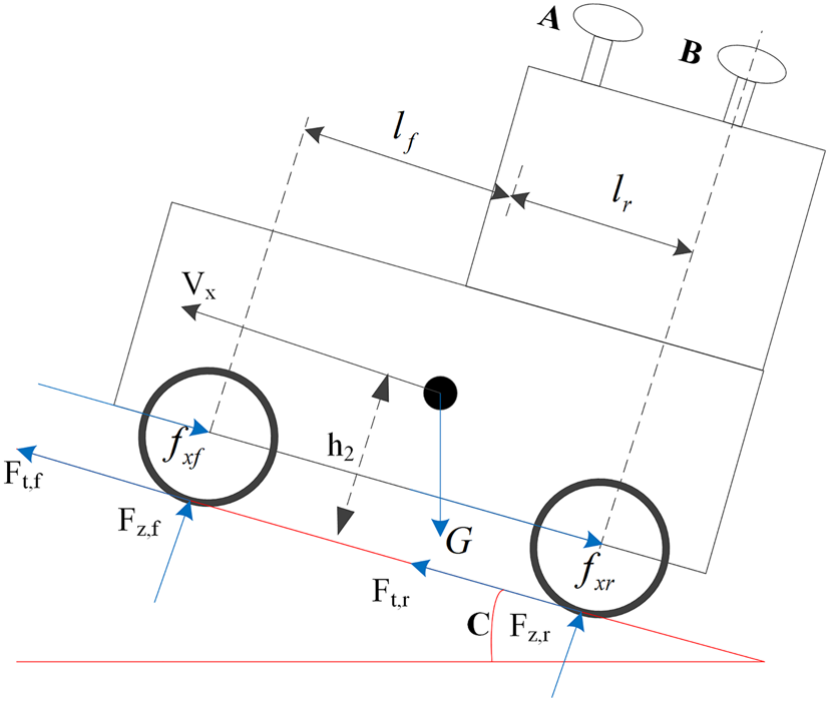
Force analysis diagram of a tractor.

When a tractor is running on sloping ground, assuming that the air resistance is ignored, the force analysis diagram of the tractor can be simplified into Fig. 4.

According to Newton’s second law of motion, the force balance along the slope direction can be expressed in the following form:

17 $$F_{tf} + F_{{{\text{tr}}}} - f_{xf} - f_{xr} - G\sin c = m\dot{V}_{x} .$$

Rajamani^18^ defines the slip rate as:

18 $$s = \frac{{u_{w} - \omega_{w} r_{r0} }}{{u_{w} }} \times 100\% ,$$

where *u*_*w*_ represents the speed of the wheel centre, *ω*_*w*_ represents the wheel angular velocity, and *r*_*r0*_ represents the wheel radius when the tractor is not braking.

When the slip ratio *s* is small, *F*_*tf*_ and *F*_*tr*_ have a linear relationship with *s*, which can be approximately expressed as:

19 $$\left\{ {\begin{array}{*{20}l} {F_{tf} = C_{f} \times s_{f} } \\ {F_{tr} = C_{r} \times s_{r} } \\ \end{array} } \right..$$

The rolling resistance of the front and rear wheels is approximated by the following formula:

20 $$\left\{ {\begin{array}{*{20}l} {f_{xf} = k \times F_{zf} } \\ {f_{xr} = k \times F_{zr} } \\ \end{array} } \right.,$$

where *k* represents the rolling resistance coefficient.

When the rear wheel is the moment balance point Σ*M*_*tire-r*_ = 0, the moment equation is:

21 $$F_{zf} \times (l_{f} + l_{r} ) - m\dot{V}_{x} h_{2} + Gh_{2} \sin C - Gl_{f} \cos C = 0.$$

From the above equation, we can obtain:

22 $$F_{zf} = \frac{{m\dot{v}h_{2} - Gh_{2} \sin C + Gl_{f} \cos C}}{{l_{f} + l_{r} }}.$$

In the same way

23 $$F_{zr} \times (l_{f} + l_{r} ) - m\dot{V}_{x} h_{2} - Gh_{2} \sin C - Gl_{r} \cos C = 0,$$

24 $$F_{zr} = \frac{{ - m\dot{V}_{x} h_{2} + Gh_{2} \sin C + Gl_{r} \cos C}}{{l_{f} + l_{r} }}.$$

We can see that the acceleration of the vehicle $\dot{V}_{x}$ during driving will affect the support of the ground on the tires *F*_*z*_.

### Model of a tractor electrohydraulic steering system

The electronically controlled hydraulic steering system of a tractor can be regarded as a mathematical model with a reversing valve control voltage *U*(*s*) as input and a steering angle *δ*(*s*) as output (Shinji and Tsugiharu^19^). When considering the actual delay in the system, the open-loop transfer function of the hydraulic steering system can be expressed as

25 $$G(s) = \frac{\delta (s)}{{U(s)}} = \frac{K}{s(\tau s + 1)}e^{ - Ls} .$$

The parameters are determined through tests to be *K* = 22.7, *τ* = 0.05, and *L* = 0.12.

26 $$G(s) = \frac{\delta (s)}{{U(s)}} = \frac{22.7}{{s(0.05s + 1)}}e^{ - 0.12s} .$$

## Research on the control strategy of an autonomous tractor

**Figure 5:**
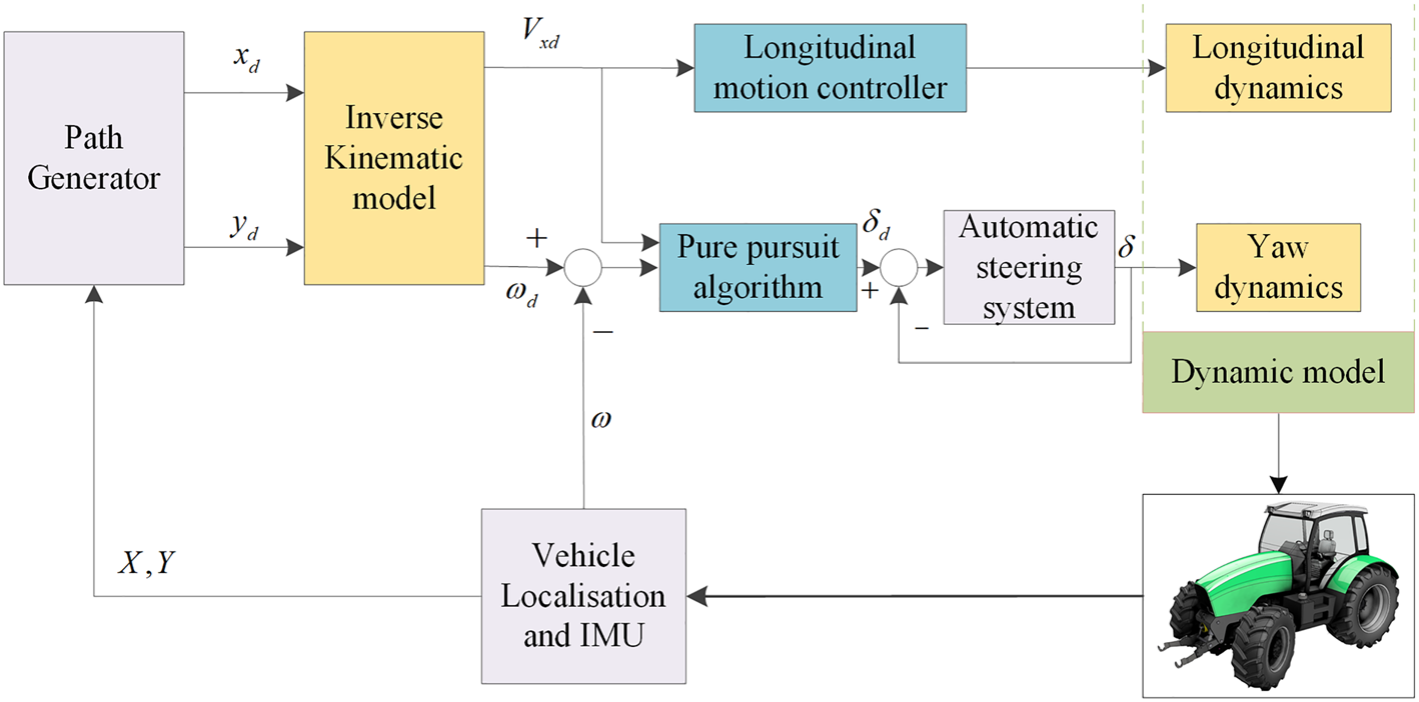
Block diagram of the overall system control scheme.

When an autonomous tractor is operating, the current position and motion state are determined in real time through satellites and compared with the target point on the set path. The steering angle of the front wheels and the longitudinal speed are controlled through correlated algorithms so that the tractor can drive along the set path. The tractor’s automatic navigation system corrects the front wheel steering angle and longitudinal speed to make the vehicle drive along the set path. The overall control block diagram of a tractor is shown in Fig. 5.

As shown in Fig. 5, the autonomous tractor host computer system calculates the deviation between the current position of the tractor and the set path through the corresponding path generation algorithm and calculates the desired longitudinal speed and yaw rate through the corresponding kinematics model. The longitudinal motion controller controls the longitudinal speed of the tractor, and the pure pursuit algorithm calculates the desired front wheel steering angle based on the desired yaw rate. The operation of the steering operation is completed through the automatic steering system. In this way, the tractor can drive along the set path through decoupling control of the tractor's lateral movement and longitudinal movement.

### Longitudinal and lateral motion control method

To control the longitudinal and lateral motion of a tractor, it is necessary to study the longitudinal speed and the steering angle of the front wheels and determine the desired longitudinal speed and the desired yaw rate from the tractor inverse kinematics model obtained above.

For longitudinal speed control, this study uses a PID control strategy, and for the control of the front wheel steering angle, this study uses a pure pursuit algorithm to determine the desired front wheel angle according to the desired longitudinal speed and the desired yaw rate. Finally, path tracking is completed by decoupling the control of longitudinal movement and the lateral movement.

### Lateral tracking control strategy

**Figure 6:**
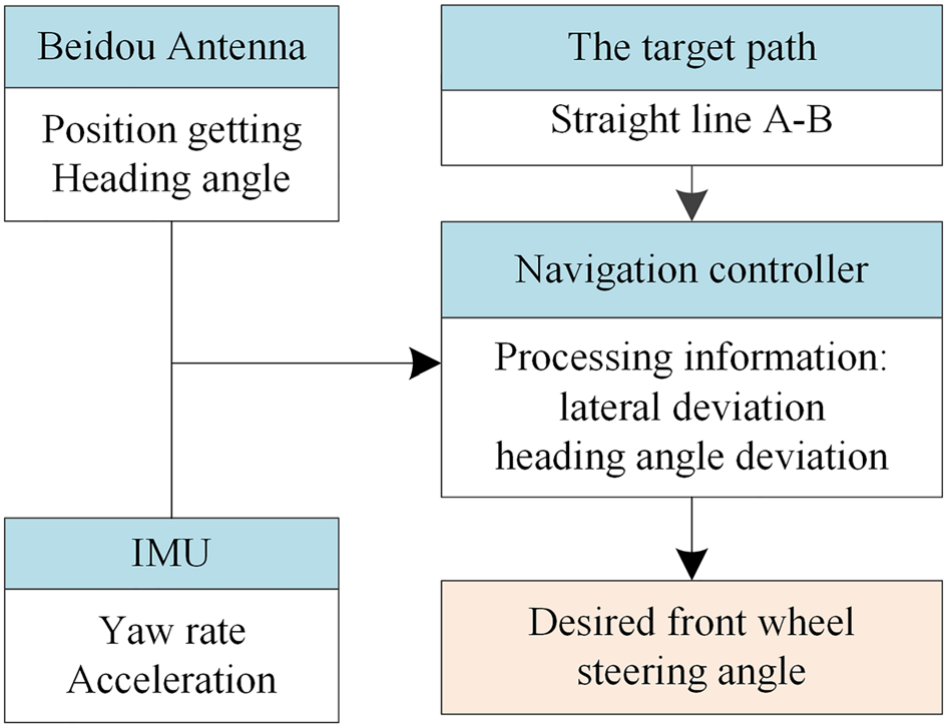
Block diagram of the desired front wheel steering angle generation.

To enhance the positioning accuracy of tractors, inertial navigation is usually used to assist satellite positioning^20^. The navigation controller calculates the lateral deviation and heading angle deviation based on the position information and path information and generates the expected front wheel steering angle, as shown in Fig. 6.

In the path tracking algorithm, the pure pursuit algorithm does not rely on complex mathematical models. It is used to calculate the arc required by the vehicle to track the target path. The test vehicle must follow the calculated arc to move from its current position to a target position at the next moment, and the target point is selected from the set path. The pure pursuit algorithm continuously iterates, and the preview point P continuously updates the position along the path so that the vehicle travel path forms a smooth tracking trajectory and finally makes the vehicle travel trajectory completely coincide with the path A–B, completing the tracking control^21,22^.

**Figure 7:**
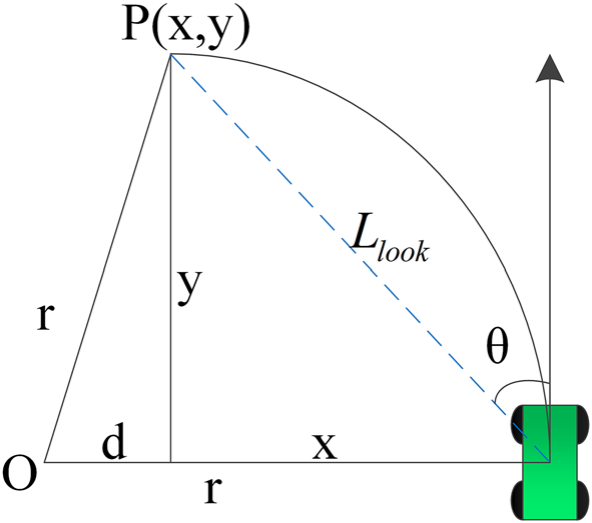
Geometric relationship of the pure pursuit algorithm.

The geometric relationship of the pure pursuit algorithm is shown in Fig. 7.

In Fig. 7, r is the trajectory radius, O is the centre of rotation, x is the deviation of the preview point P relative to the lateral coordinate of the car body, y is the deviation of the preview point P relative to the longitudinal coordinate of the tractor body, point P is a suitable point of the set path, the distance between point P and the vehicle body is the preview distance *L*_*look*_, and *θ* is the heading deviation. Then, the following formula can be obtained from the geometric relationship in the above figure:

27 $$x^{2} + y^{2} = L_{look}^{2} ,$$

28 $$d^{2} + y^{2} = r^{2} ,$$

29 $$d + x = r.$$

From the above equation

30 $$r = \frac{{{\text{L}}_{look}^{2} }}{2x}.$$

The path curvature when turning is *k*, *k* = 1/*r*. Then, the above equation can be written as:

31 $$k = \frac{2x}{{{\text{L}}_{look}^{2} }}.$$

The following geometric relationship is obtained from Fig. 7.

32 $$\sin \theta = \frac{x}{{{\text{L}}_{look} }}.$$

When the heading deviation angle *θ* is small ($\theta \to 0$), sin* θ* ~ *θ*. Equation (32) can be approximately expressed as:

33 $$\theta \approx \frac{x}{{{\text{L}}_{look} }}.$$

The path curvature can be approximately expressed as:

34 $$k = \frac{2\theta }{{{\text{L}}_{look} }}.$$

It can be seen from the above formula that the pure pursuit algorithm is only related to the preview distance *L*_*look*_.

The output of the pure pursuit algorithm is the curvature of the path when the tractor is driving, and the curvature can be converted into the yaw motion and longitudinal motion of the tractor.

We can get obtain *ω* from Eq. (35).

35 $$k = \frac{\omega }{V},$$

where the curvature *k* = 1/*r*, and the above formula can be written as:

36 $$\omega = V \times k = \frac{V}{r}.$$

The values of the *ω* and *V* when driving on a curve are used to control the steering angle of the front wheels and the speed of the vehicle, respectively.

**Figure 8:**
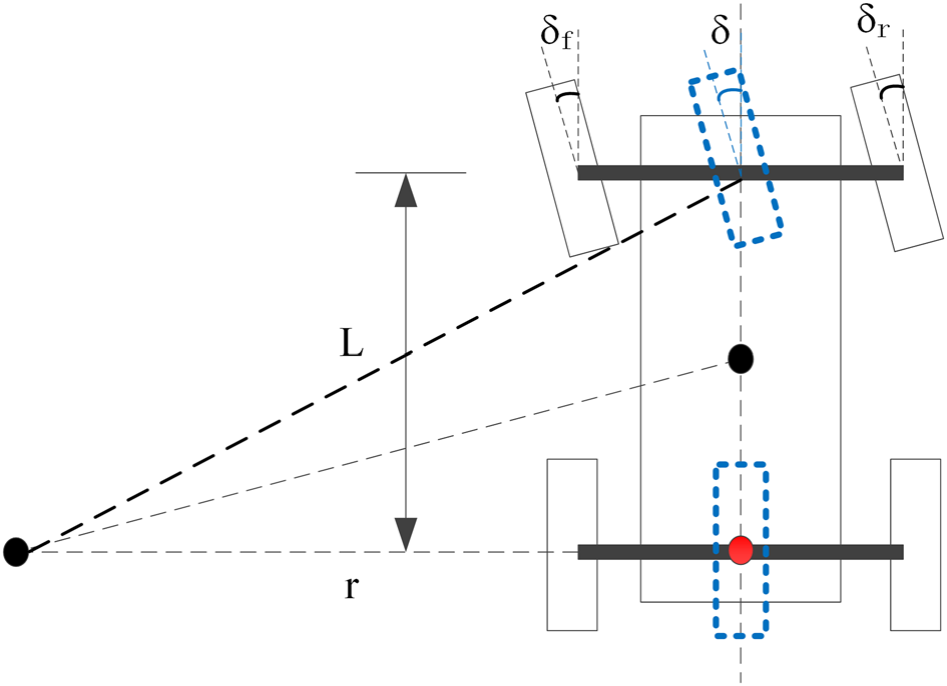
Ackerman steering geometry diagram.

According to Fig. 8, Gillespie^23^ expressed the function of the path curvature radius as

37 $$\delta = \frac{L}{r},$$

where

38 $$r = \frac{1}{k} = \frac{V}{\omega }.$$

Therefore, the desired front wheel angle is denoted as:

39 $$\delta = \frac{\omega L}{V}.$$

### Hydraulic steering system control strategy

**Figure 9:**
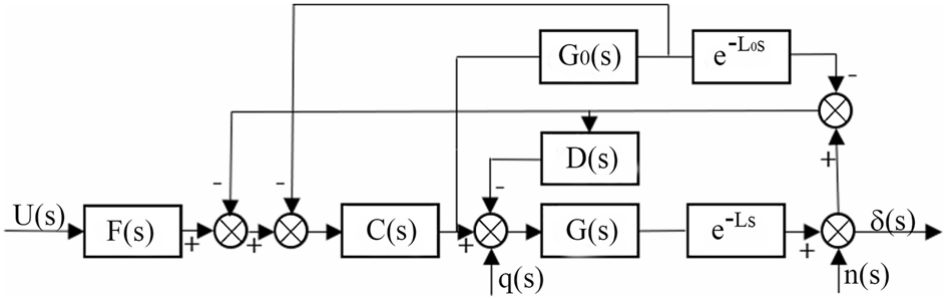
Structural block diagram of hydraulic steering control.

From the hydraulic steering system model established in the previous section, the steering system is a second-order integral time-delay object, while the PID method has poor anti-interference performance and poor tuning effect, so when the steering system is disturbed, it is easy to produce oscillation and divergence phenomena, resulting in poor adaptability of the steering system^24^. In actual industrial production, many controlled objects have pure delay characteristics. To improve the delay in industrial control links, Smith^25^ proposed a compensation model. The Smith predictive controller can better improve the pure delay link of the system, but it is necessary to establish a sufficiently accurate control object model, and the anti-interference ability is poor. Therefore, it is very meaningful to improve the traditional Smith predictive controller^26^. This study proposes a two-degree-of-freedom control scheme based on the improved Smith predictive control to improve the stability and anti-interference of the automatic steering system and reduce unnecessary additional steering. The block diagram of the proposed control scheme is shown in Fig. 9. In the figure, C(s) is the tracking controller, which is used to track the control voltage signal. The filter controller F(s) eliminates the overshoot in the automatic control process. D(s) is a disturbance suppression controller, which is used to suppress disturbances in the control system.

### Design of the tracking controller C(s)

When the actual steering system and the steering system model match each other (nominal case), the actual object is equal to the object model, that is, *G*(*s*)*e*^−Ls^ = *G*_*0*_(*s*)*e*^*−L0s*^. The tracking response transfer function from reference signal *U*(*s*) to system output *δ*(*s*) can be obtained from Fig. 9.

40 $$Y(s) = \frac{\delta (s)}{{U(s)}} = F(s)\frac{{G_{0} (s)C(s)}}{{1 + G_{0} (s)C(s)}}.$$

The transfer function from the input interference signal *q*(s) to the system output *δ*(*s*) is

41 $$Y_{q} (s) = \frac{\delta (s)}{{q(s)}} = \frac{{G_{0} (s)\left[ {1 + G_{0} (s)C(s)(1 - e^{{ - L_{0} s}} )} \right]e^{{ - L_{0} s}} }}{{(1 + G_{0} (s)C(s))(1 + D(s)G_{0} (s)e^{{ - L_{0} s}} )}}.$$

It can be seen from the above formula that the input response and interference response in the control structure are completely decoupled, and the parameters of the controller can be obtained according to the internal model control method. Therefore, the IMC-PID controller is adopted to design the tracking controller and disturbance suppression controller. D(s) does not affect the input response, and it is used to stabilize time-delay unstable objects. The stability of the system is related to the characteristic equation $(1 + G_{0} (s)C(s))(1 + D(s)G_{0} (s)e^{{ - L_{0} s}} )\; = \;0$ and is mainly used to stabilize $1 + D(s)G_{0} (s)e^{{ - L_{0} s}}$.

**Figure 10:**
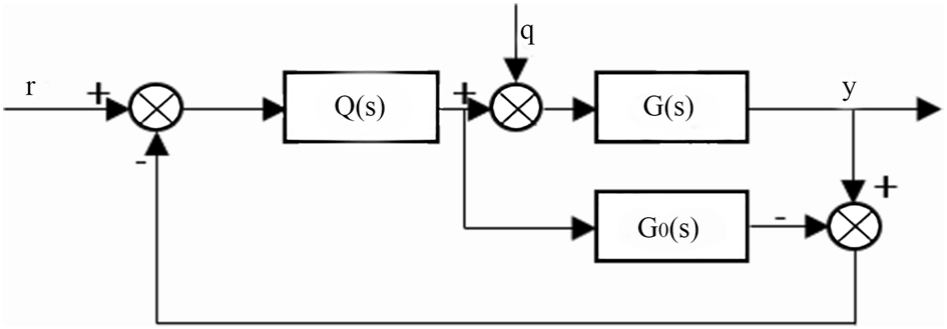
Structure of the internal model control principle.

**Figure 11:**
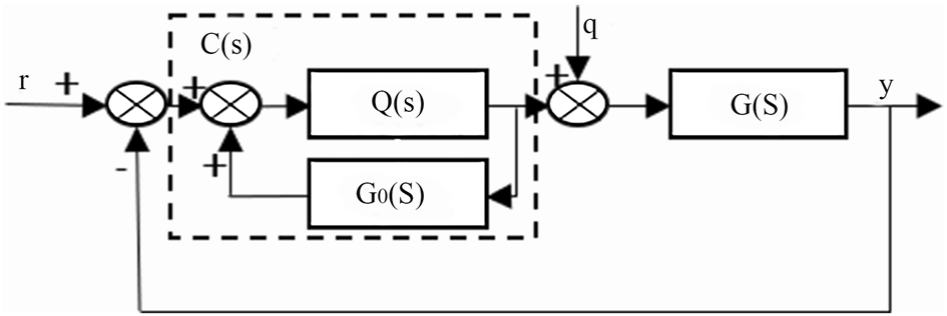
Structure of the equivalent feedback control system.

Figures 10 and 11 show the internal model control principle structure and the equivalent feedback control system structure.

Through the structure of the internal model control principle, the following formula can be obtained:

42 $$\frac{y}{r} = \frac{C(s)G(s)}{{1 + C(s)G(s)}},$$

43 $$\frac{y}{{\text{q}}} = \frac{G(s)}{{1 + C(s)G(s)}}.$$

The internal model controller and feedback controller are

44 $$Q(s) = G_{0 - }^{ - 1} (s)f(s),$$

45 $$C(s) = \frac{Q(s)}{{1 - Q(s)G_{0} (s)}}.$$

According to the relevant theory of internal model control^27^, the integral link is approximated by the first-order link, and when *α* is taken as a large enough number

46 $$\frac{K}{s} \approx \frac{\alpha K}{{\alpha s + 1}}.$$

The process model is expressed as

47 $$G_{0} (s)e^{ - Ls} = \frac{K}{s(\tau s + 1)}e^{ - Ls} \approx \frac{\alpha K}{{(\alpha s + 1)(\tau s + 1)}}e^{ - Ls} .$$

According to the design conditions of the internal model controller.

There is no unstable pole in *Q*(*s*); *Q*(*s*)*G*(*s*) is stable; (1 − *Q*(*s*)*G*(*s*)) *G*(*s*) is stable.

Therefore, the filter design is

48 $$f(s) = 1/(\lambda s + 1).$$

In this case, controller *C*(*s*) is represented by Eq. (45)

49 $$C(s) = \frac{{(\alpha s + 1){{(\tau s + 1)} \mathord{\left/ {\vphantom {{(\tau s + 1)} {\alpha K}}} \right. \kern-\nulldelimiterspace} {\alpha K}}}}{{(\lambda s + 1 - e^{ - Ls} )}}.$$

The first-order Taylor expression is adopted to approximate the delay term $e^{ - Ls} \approx 1 - Ls$, and an IMC-PID controller is obtained

50 $$C(s) = \frac{\alpha + \tau }{{\alpha K(\lambda + L)}}\left( {1 + \frac{1}{(\alpha + \tau )s} + \frac{\alpha \tau }{{(\alpha + \tau )}}s} \right),$$

where the corresponding parameter of the controller is

$$K_{p1} = \frac{\alpha + \tau }{{\alpha K(\lambda_{1} + L)}};\;T_{i1} = \alpha + \tau ;\;T_{d1} = \frac{\alpha \tau }{{\alpha + \tau }}.$$

The filter *F*(*s*) is designed to be

51 $$F(s) = \frac{1}{{T_{i} s + 1}}.$$

#### Disturbance rejection controller D(s)

When the actual steering system and the steering system model match each other, the compensation sensitivity function of the load interference suppression closed-loop is expressed as

52 $$S(s) = \frac{G(s)D(s)}{{1 + G(s)D(s)}}.$$

The form of the above formula is similar to that of the *Y*(*s*) formula, so *D*(*s*) is designed with reference to the knowledge related to internal model control theory, similar to *C*(*s*).

53 $$\frac{D(s)G(s)}{{1 + D(s)G(s)}} = G(s)Q(s).$$

In the above formula, *Q*(*s*) is used to represent the internal model controller. Then, referring to the relevant knowledge of the internal model controller^27^, *Q*(*s*) is expressed as

54 $$Q(s) = G_{0 - }^{ - 1} (s)f(s) = \frac{(\alpha s + 1)(\tau s + 1)}{{\alpha K}}\frac{1}{(\lambda s + 1)}.$$

From Eq. (53), the following formula can be obtained:

55 $$D(s) = \frac{Q(s)}{{1 - Q(s)G_{0} (s)}} = \frac{{(\alpha s + 1){{(\tau s + 1)} \mathord{\left/ {\vphantom {{(\tau s + 1)} {\alpha K}}} \right. \kern-\nulldelimiterspace} {\alpha K}}}}{{(\lambda s + 1 - e^{ - Ls} )}}.$$

The disturbance rejection controller formula is further approximated by first-order Taylor to obtain the following PID form:

56 $$D(s) = \frac{\alpha + \tau }{{\alpha K(\lambda + L)}}\left( {1 + \frac{1}{(\alpha + \tau )s} + \frac{\alpha \tau }{{(\alpha + \tau )}}s} \right),$$

where

$$K_{p2} = \frac{\alpha + \tau }{{\alpha K(\lambda_{2} + L)}};\;T_{i2} = \alpha + \tau ;\;T_{d2} = \frac{\alpha \tau }{{\alpha + \tau }}.$$

#### Hydraulic steering control system simulation

According to the model parameter, *K* = 22.7, *τ* = 0.05, and *L* = 0.12 of the hydraulic steering system determined in the previous section, the values of the two controllers *C*(*s*) and *D*(*s*) are established. Since the process is not clear and unknown, take $\lambda_{1} = 0.06,\lambda_{2} = 0.18,\alpha_{1} = 0.13,\alpha_{2} = 0.55$ to obtain the following form of the controller:

$$F(s) = 1/(0.18s + 1),$$

$$C(s) = 0.338\left(1 + \frac{1}{0.18s} + 0.036\;{\text{s}}\right),$$

$$D(s) = 0.203\;\left( {1 + \frac{1}{0.6s} + 0.0458\;{\text{s}}} \right).$$

**Figure 12:**
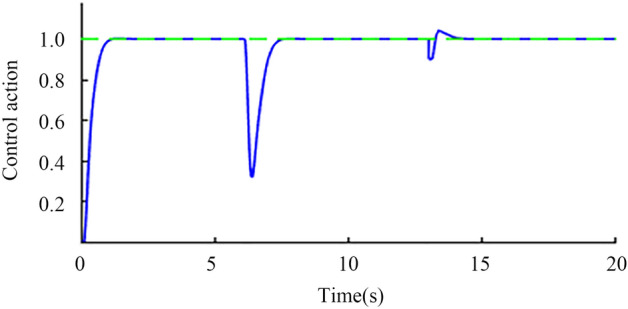
Nominal response at step signal input.

**Figure 13:**
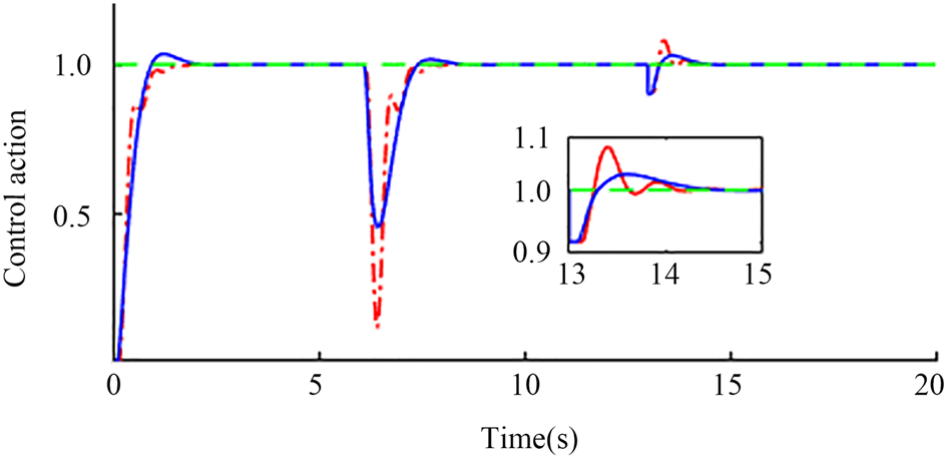
Perturbation response when the step signal is input.

Step signal input simulation. When the time is 0 s, a unit step signal is added to the input of the set point. When the time is 6 s and 13 s, the step interference signals with the amplitudes of 0.2 and 0.1 are added to the input and output of the controlled object, respectively. The output waveform shown in Fig. 12 is obtained through a Simulink Scope. It can be seen from the figure that the set value response of the system has no overshoot and has good anti-interference ability. In addition, to verify the robustness of the system, corresponding assumptions are made for the delay time and gain constant of the actual object. Assuming that the two are reduced by 20% and increased by 20%, the perturbation system obtained the results shown in Fig. 13 through a Simulink Scope. The output waveform, the blue solid line, is the response of the perturbation system when both parameters are reduced by 20%, and the red solid line is the response of the perturbation system when both parameters are increased by 20%. The red and blue curves in the figure can quickly return to a stable state after being disturbed, which proves the robust performance of the system.

**Figure 14:**
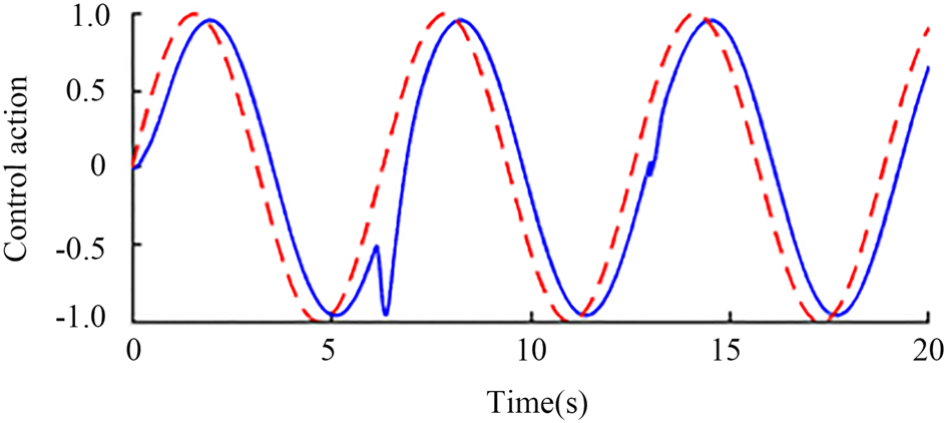
Nominal response when sinusoidal signal is input.

**Figure 15:**
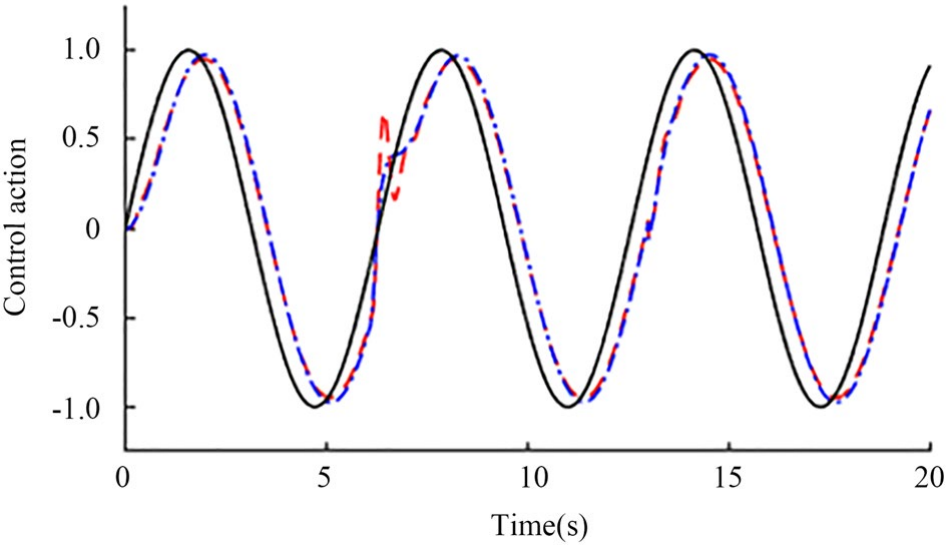
Perturbation response when a sinusoidal signal is input.

Sinusoidal signal simulation. When the time is 0 s, the unit sine signal is added to the input of the set point. When the time is 6 s and 13 s, the input and output of the controlled object, respectively, are added with step interference signals with amplitudes of 0.2 and 0.1 in the opposite direction. Finally, the output waveform shown in Fig. 14 is obtained through an oscilloscope. The red line is the standard sine signal, and the blue line is the nominal system response curve. After being disturbed, the blue curve can quickly recover to the stable state, which proves the anti-interference of the system. To verify the robustness of the system, corresponding assumptions are made for the delay time and gain constant of the actual object. Assuming that the two are reduced by 20% and increased by 20%, the perturbation system obtained the output waveform as shown in Fig. 15 through an oscilloscope, where the blue line is the response of the perturbation system when both parameters are reduced by 20%, and the red line is the response of the perturbation system when both parameters are increased by 20%. The red and blue curves can quickly recover to the stable state after being disturbed, which proves the robust stability of the system.

**Figure 16:**
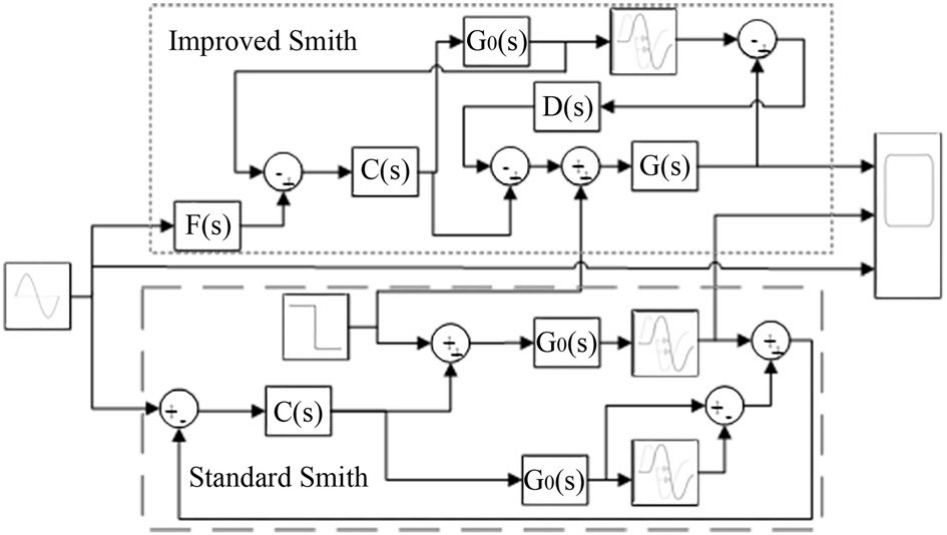
Improved Smith and standard Smith predictive control model diagram.

To test the effect of improving Smith’s predictive control, the model shown in the Fig. 16 was built.

**Figure 17:**
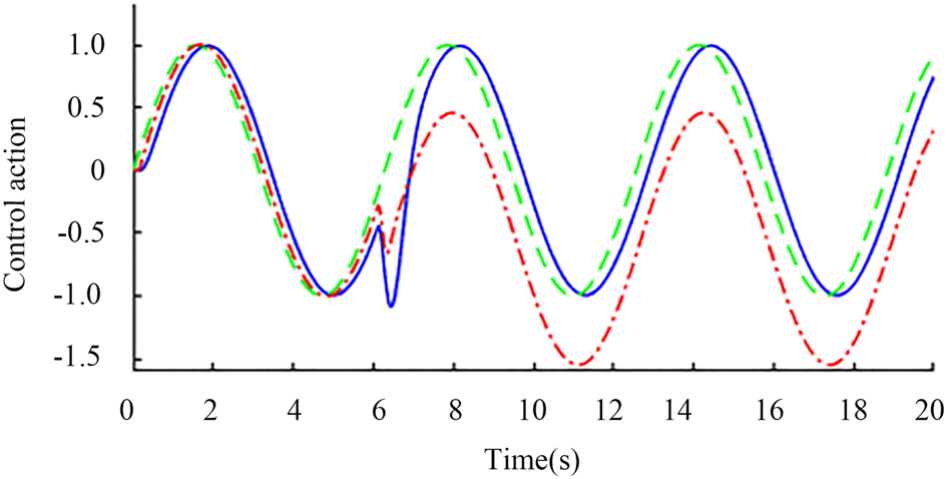
Smith predictive control comparison simulation diagram.

The results shown in Fig. 17 are obtained through simulation.

The green dotted line in Fig. 17 shows the sine signal input unit, the blue solid line shows the improved Smith prediction control, and the red dotted shows the line standard Smith prediction control. Figure 17 shows that when 6 s of external disturbance is input, the improved Smith predictive control quickly recovers to a stable state in approximately 7 s, and the tracking effect is significantly better than that of the standard Smith predictive control.

It can be seen from the above simulation diagram that the hydraulic steering control method used in this paper not only improves the anti-interference ability of the system but also makes the system have strong robust stability. In the hydraulic steering system control scheme proposed based on the improved Smith predictive control theory, the disturbance suppression response and tracking response of the steering system are independent of each other. The two controllers are designed in IMC-PID form, and they can be independently adjusted and optimized through a single parameter. Based on ensuring the performance of the setpoint tracking response, the system has good anti-interference ability and can fully meet the requirements of the actual work of the autonomous tractor.

### Tractor longitudinal motion control strategy

Considering the operation situation of a tractor, the longitudinal dynamics model can be simplified to a first-order system^28,29^. Therefore, the longitudinal motion system model of the tractor is represented by “Longitudinal dynamics model of a tractor” section

57 $$G\left( s \right) = \frac{{V_{x} }}{{V_{xd} }} = \frac{K}{Ts + 1},$$

where *V*_*xd*_ represents the desired speed of the tractor, *V*_*x*_ represents the actual speed of the tractor, and the parameters of *K* and *T* were estimated as *K* = 1.04 and *T* = 2.06, respectively, by related experiments.

58 $$G\left( s \right) = \frac{1.04}{{2.06s + 1}}.$$

In the longitudinal motion, a PID controller is used to control the speed of the vehicle. PID parameters are selected according to the longitudinal motion system model, and finally *K*_p_ = 6, *K*_i_ = 3, *K*_d_ = 1.5

## System simulation and testing

In this section, a system simulation was executed on the vehicle dynamics simulation software CarSim, in connection with MATLAB/Simulink to test the performance of the proposed control method^30^. Simulink was used in the simulation to build the model. The time step was fixed-step: 0.025 s, and auto (Automatic Solver Selection) was set by Solver. The time step of CarSim was 0.001 s, and the output file time step was 0.025 s with the integration method: AM-2 (2 updates per step).

**Figure 18:**
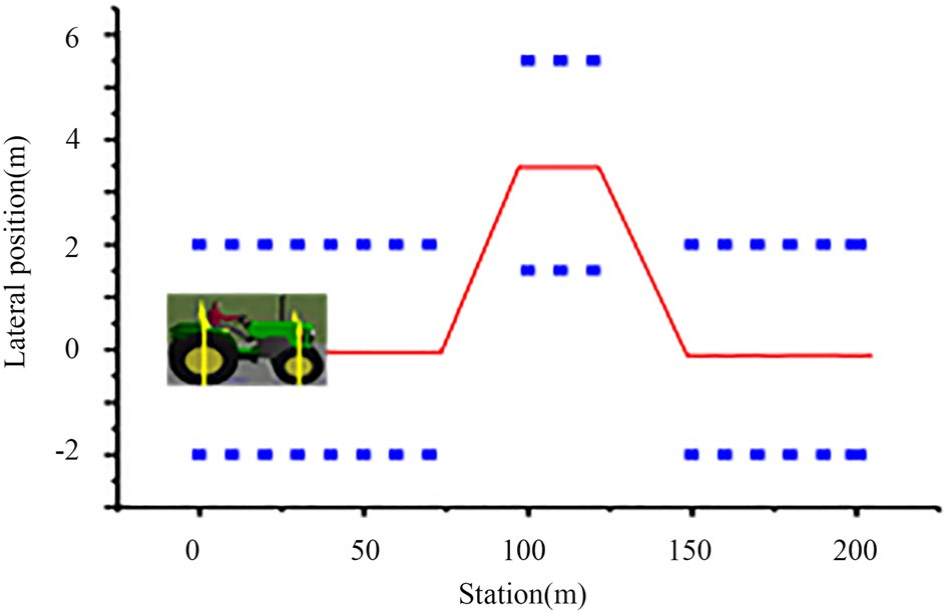
Moose test track.

The tractor model was selected from CarSim. The tractor parameters are given in Table 2. The target speed of the vehicle was 30 km h^−1^, and the constant value of the tire-road friction coefficient (u) was 0.85. The simulation was path tracking on a Moose test track (Fig. 18). The reference (red line) is a double line change, which is suitable to check the controller’s performance.

**Table 2:** Parameters of the tractor model.

Name	m	*I*_*z*_	*l*_*f*_	*l*_*r*_	*C*_*f*_	*C*_*r*_
	kg	kg m^2^	m	m	N rad^−1^	N rad^−1^
Value	3000	1765	1.05	1	− 80,000	− 90,000

**Figure 19:**
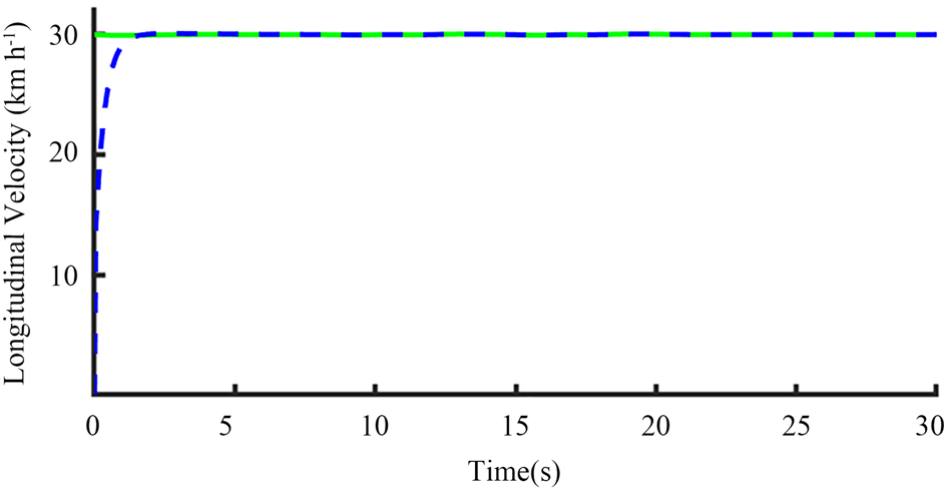
Graph of the desired and actual longitudinal velocity at the CG.

**Figure 20:**
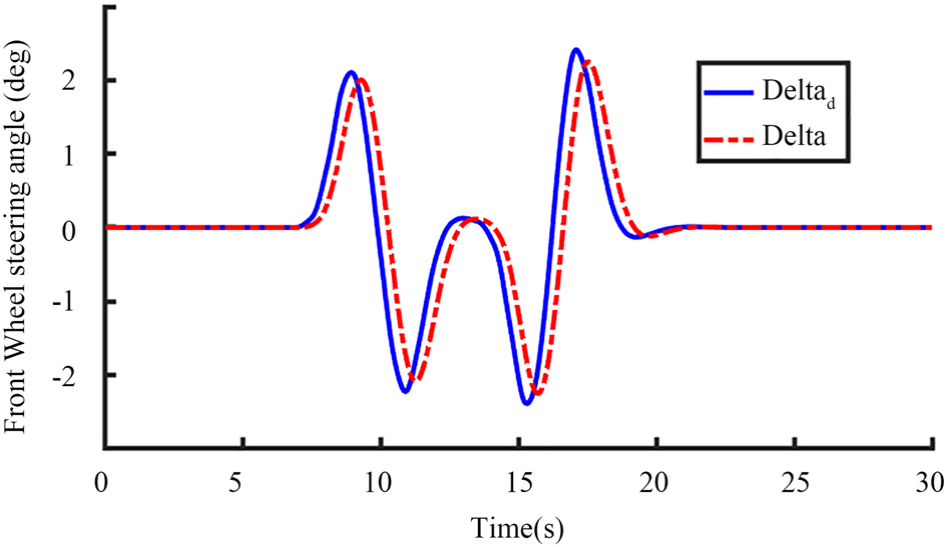
Desired front wheel angle and actual front wheel angle change with time.

**Figure 21:**
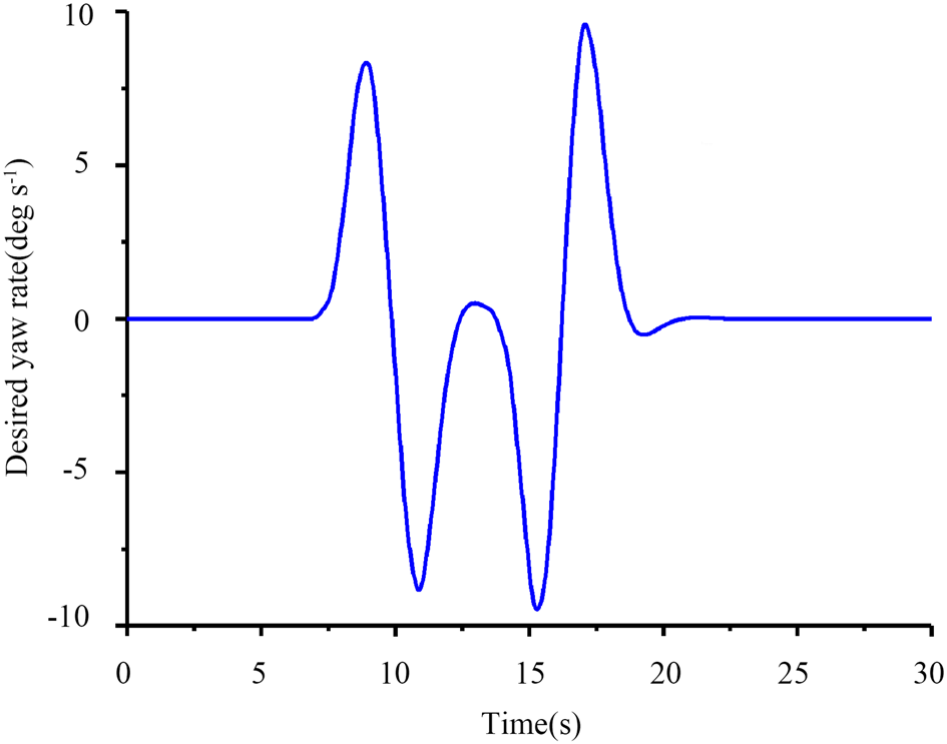
Graph of the desired yaw rate versus time.

**Figure 22:**
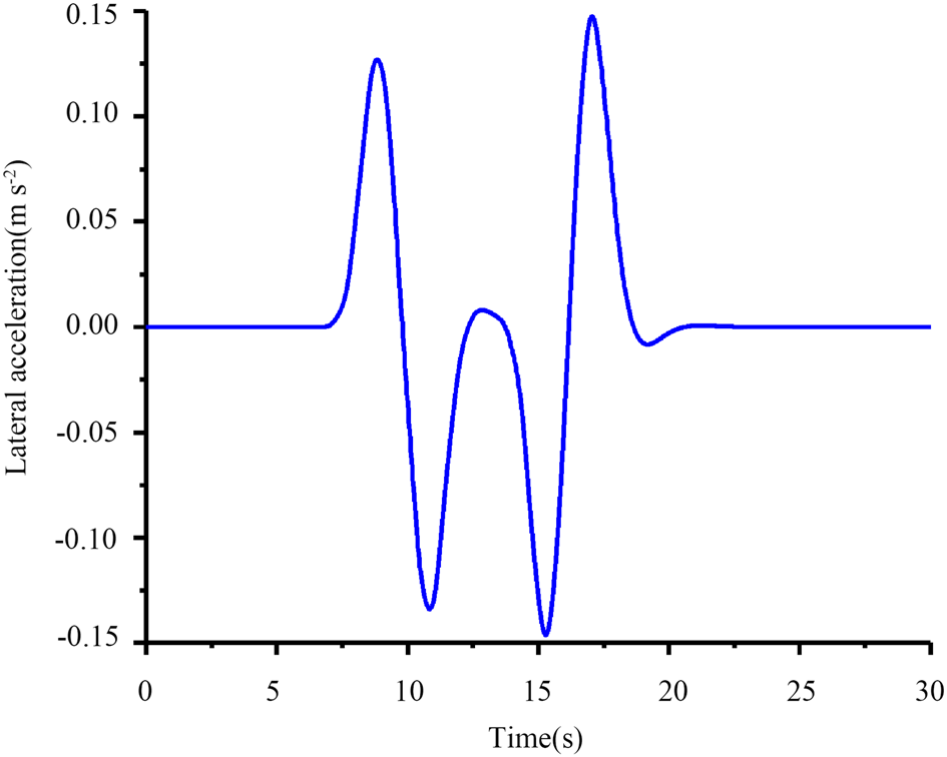
Lateral acceleration at the CG.

**Figure 23:**
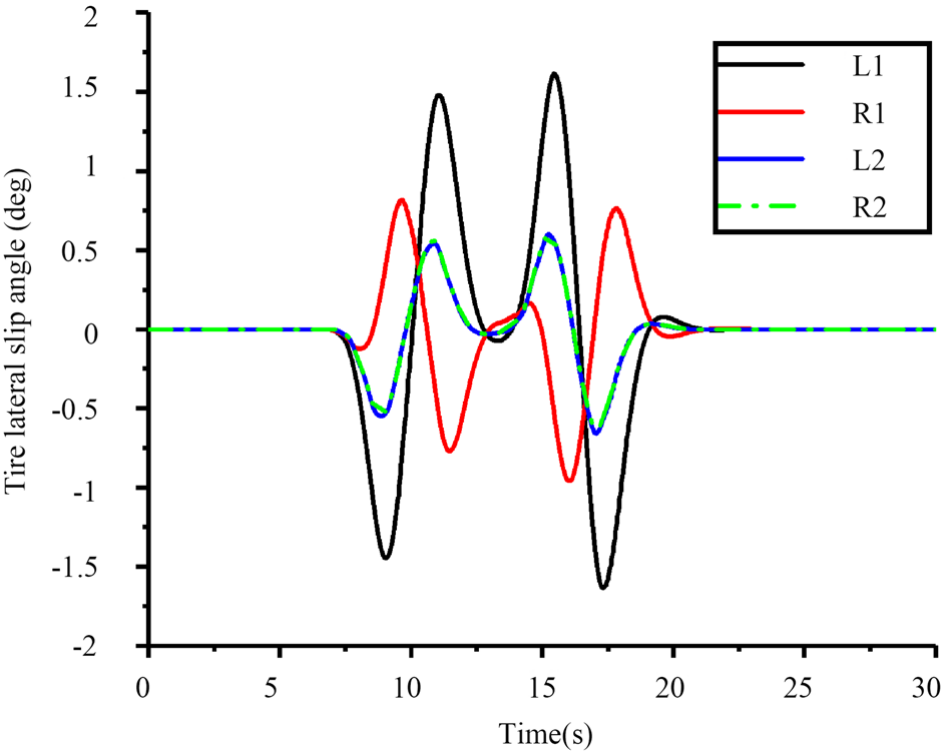
Tire lateral slip angle.

**Figure 24:**
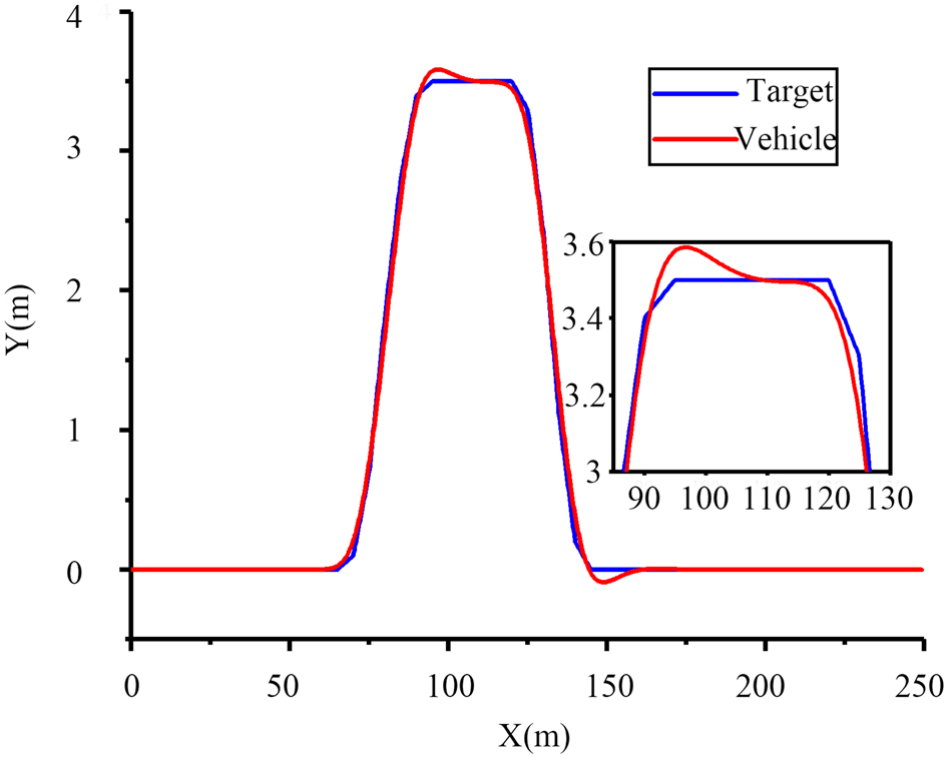
Tracking effect at 30 km h^−1^.

The overall CarSim and Simulink simulation model was run, and the relevant simulation results are shown in Figs. 19, 20, 21, 22, 23 and 24.

In Fig. 19, the green line is the expected speed of 30 km h^−1^, and the blue line is the actual speed change curve of the tractor. When the expected speed information is sent to the longitudinal motion controller, the tractor can quickly respond to it, and finally the actual vehicle speed is consistent with the expected speed, indicating that the tracking effect of longitudinal speed meets the demand.

In Fig. 20, the blue curve is the desired front wheel angle, and the red curve is the actual front wheel angle. The figure shows that the automatic steering system can quickly respond to the desired steering information sent by the host computer system, and the time delay between the actual steering and the desired steering is in line with the actual working conditions.

It can be seen from Figs. 20 and 21 that the desired front wheel steering angle change trend is roughly the same as the desired yaw rate change trend, which is in line with actual conditions.

It can be seen from Fig. 22 that the peak value of lateral acceleration is approximately 0.15 < 0.4 g. This effectively guarantees the path tracking effect while considering the comfortable requirements and driving stability of the tractor. Figure 23 shows that the tractor’s front tire slip angles of L1 and R1 start to decrease at 7 s and then increase. At 13 s, the trends of L1 and R1 are opposite, and the trend from 7 to 20 s is in line with the reality steering operation. Generally, the front wheels of the tractor are used for steering, and the rear wheels are used for driving, so the tire slip angles of the rear tires L2 and R2 have the same tendency.

To verify the path tracking effect of the tractor, the test was carried out at a speed of 30 km h^−1^. The test results are as follows.

It can be seen from Fig. 24 that the tractor produces obvious jitter at the turn of the set path between 60 and 150 m, and can quickly return to the straight tracking state after the turn is completed near 100 m, with a maximum tracking deviation of approximately 10 cm and the overall tracking effect meets the design requirements.

**Figure 25:**
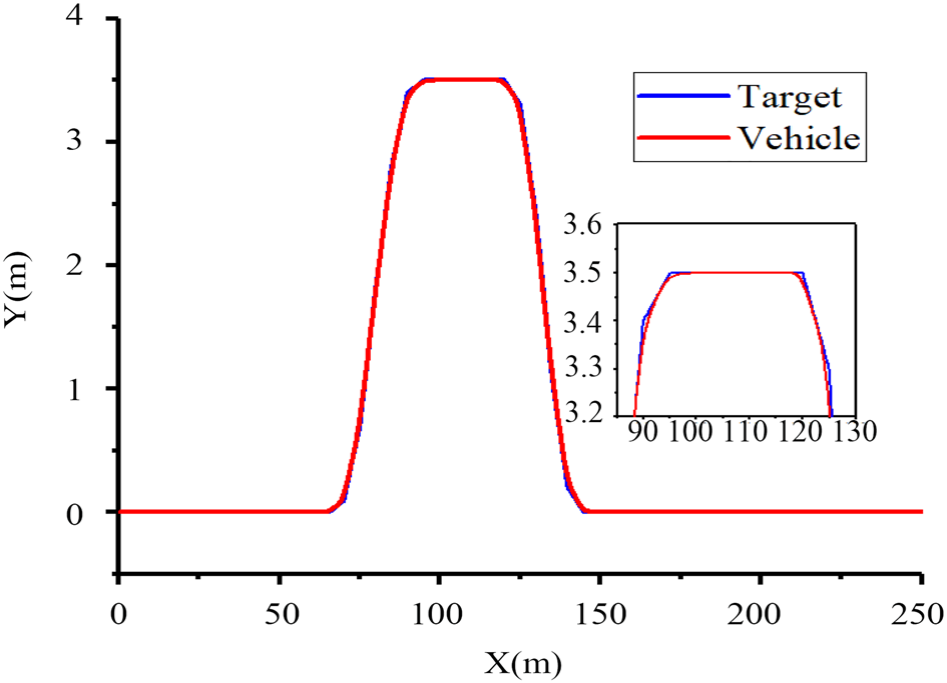
Tracking effect at 10 km h^−1^.

Figures 24 and 25 show that when the speed is reduced, the tracking effect of the tractor is significantly improved, and the operating speed of the tractor is less than 10 km h^−1^ at ordinary times, which can meet the actual operating requirements.

From Figs. 19, 20, 21, 22, 23, 24 and 25, it can be seen from the above simulation diagram that the control algorithm proposed in this paper can better control the tractor’s longitudinal and lateral movement, and make the tractor follow the set trajectory.

**Figure 26:**
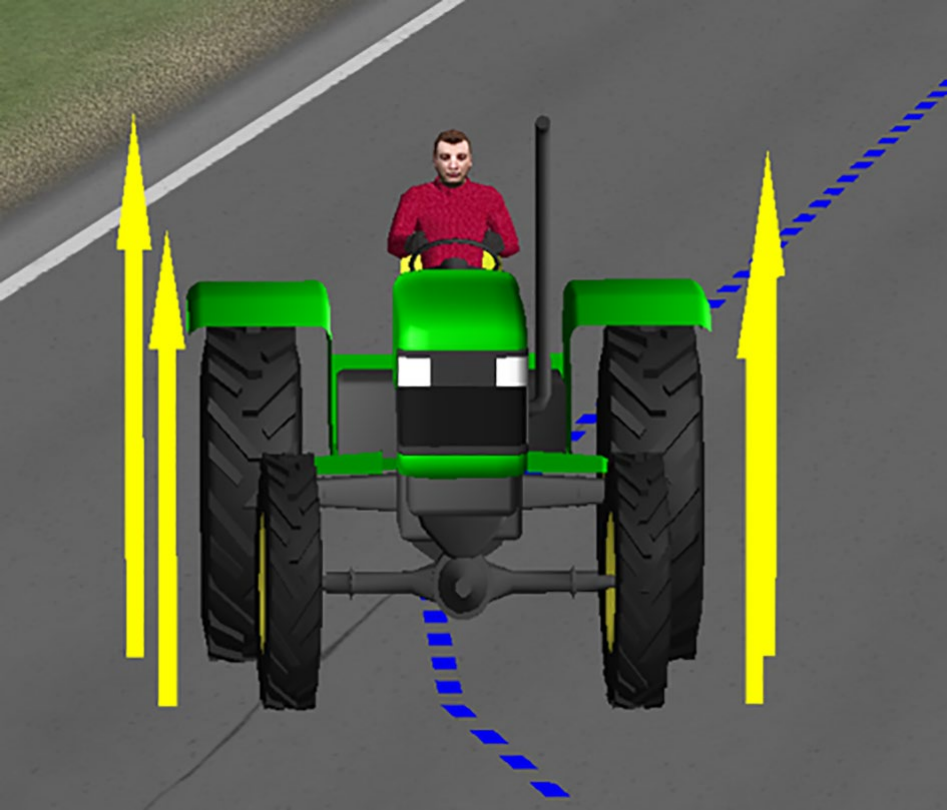
Tractor operating under road conditions.

**Figure 27:**
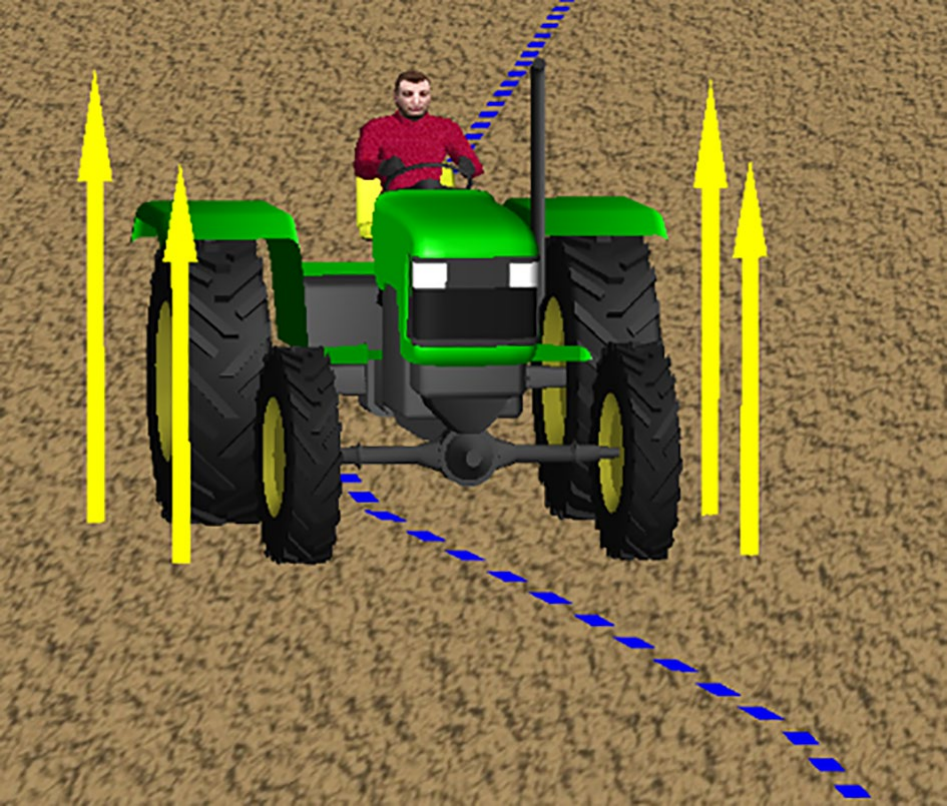
Tractor operating in farm fields.

The above test data are based on the road environment, as shown in Fig. 26. However, when the tractor is operating on farmland, the operation situation of the tractor is more complicated (as shown in Fig. 27). Hence, it is very important to study the influence of the soil friction coefficient on vehicle operation. The tire-road friction coefficient (u) of the soil was selected as 0.3, 0.35, 0.5 and 0.75 for the test to better verify the performance of the tractor control system.

**Figure 28:**
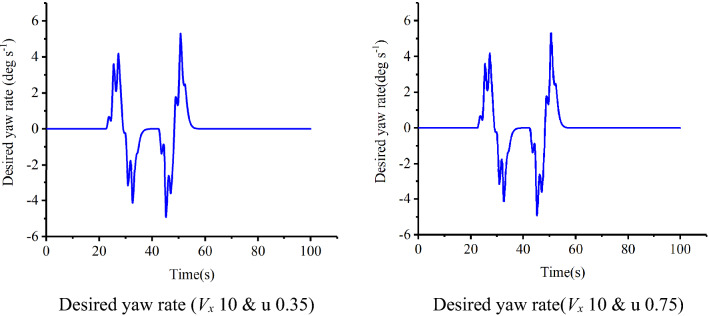
Effect of the road friction coefficient on the desired yaw rate.

When considering the influence of the road friction coefficient, the corresponding results are shown in Fig. 28.

**Figure 29:**
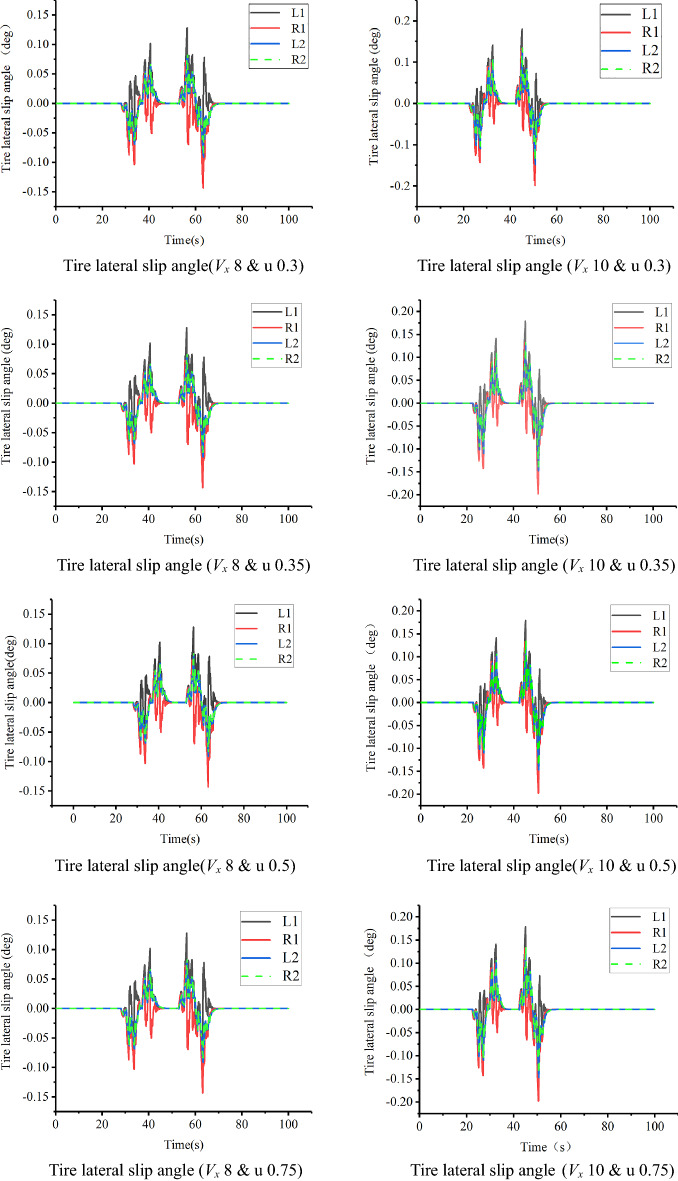
Comparison of tire lateral slip angles.

**Figure 30:**
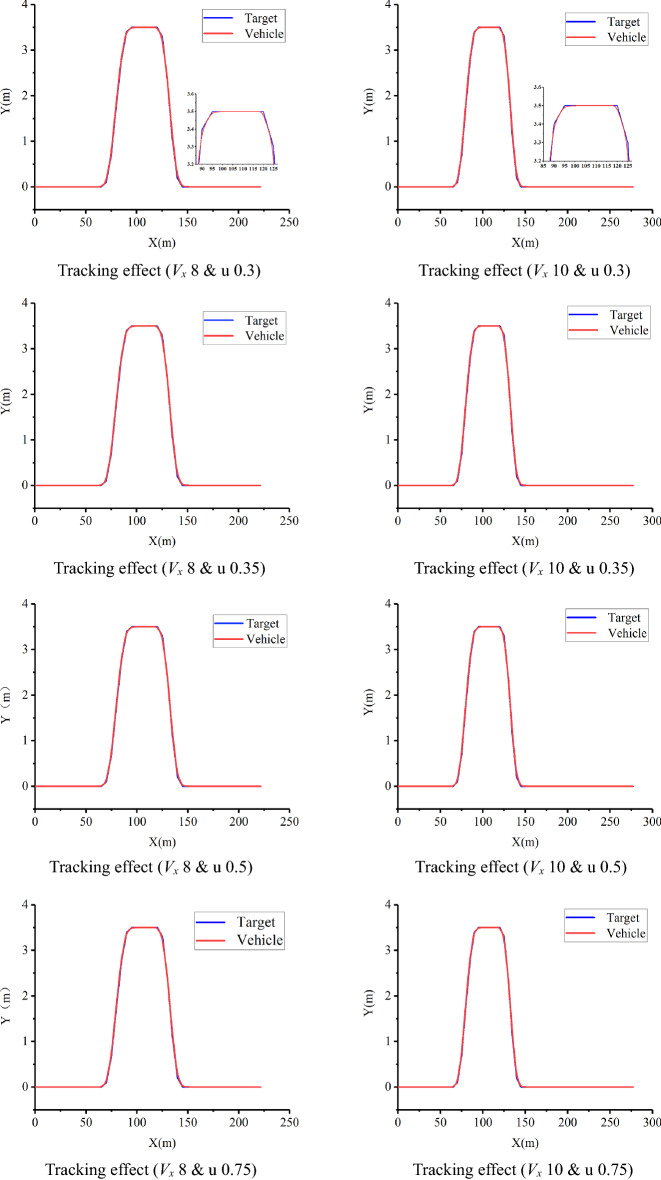
Comparison of the tracking effect.

Compared with Figs. 21 and 28, the desired yaw rate decreases significantly with the decrease in driving speed, but wobble occurs at the steering position under the influence of the soil friction coefficient. To better observe the effect of soil, the influence of different soil friction coefficients u on vehicles running under the conditions of velocities *V*_*x*_ of 8 and 10 km h^−1^ was studied, and the results are shown in Figs. 29 and 30.

As shown in Fig. 29, the tire lateral sideslip angle increases with increasing speed in the steering position. Because the lateral component of the steering position speed increases, the lateral slip angle of the tire increases, which is in line with the actual situation. By comparing Figs. 28 and 29, it can be seen that under the influence of the soil friction coefficient, the tire’s slip angle fluctuates significantly, which is in line with the changing trend of the tractor at work. The corresponding results for the path tracking effect are shown in Fig. 30.

As shown in Fig. 30, when driving at speeds of 8 and 10 km h^−1^, there is little deviation on a straight road. There will be some deviations in path tracking during the turn, but these deviations are small and have little impact on the actual operation. At the same time, it can be seen from Figs. 24, 25 and 30 that the friction coefficient of the soil has little influence on the effect of path tracking when the tractor is running at low speed, and the tracking accuracy can meet the needs of actual operation.

## Conclusion

In this paper, a path tracking control strategy for a tractor was designed based on the inverse kinematics model of the longitudinal and lateral dynamic decoupling control. The goal of controlling a tractor is to make it automatically track the desired trajectory and ensure that the tracking accuracy meets the actual agricultural production requirements. In this paper, the improved Smith predictor controller proposed for the hydraulic steering time delay verified the effectiveness of the control algorithm through simulation and comparison.

To test the overall control strategy, a joint simulation with CarSim and MATLAB/Simulink software was carried out. The experimental results show that the control strategy proposed in this paper can maintain a good tracking effect, and the driving speed can meet the actual operation requirements of the tractor. At the same time, the yaw rate, lateral acceleration, and other information when the tractor is travelling are analysed. The proposed path tracking control strategy can ensure the comfort and driving stability of the driver while ensuring the accuracy of path tracking. Considering that suspended farm tools are not considered in the vehicle dynamics modelling in this study, an overall tractor-farm tool dynamics model will be built in future research, and the tractor will be controlled according to the overall dynamics model so that the tractor can reach a better operation effect and maintain good fuel economy.

## List of symbols

*m*: Mass (kg)
*δ*: Front wheel steering angle (degrees)
*ϕ*: Heading angle (degrees)
$L$: Distance between the front and rear axle (m)
*l*_*f*_: Distance between the front axle and the CG (m)
*l*_*r*_: Distance between the rear axle and the CG (m)
*r*: Turning arc radius (m)
*V*_*x*_: Longitude velocity of the CG (m s^−^^1^)
*V*_*y*_: Lateral velocity of the CG (m s^−^^1^)
*V*_*xd*_: Desired forward velocity of the tractor (m s^−^^1^)
*F*_*lf*_: Lateral force on the front wheels (N)
*C*: Is the angle of inclination of the road on which the vehicle is travelling (degrees)
*F*_*lr*_: Lateral force on the rear wheels (N)
*F*_*tf*_: Traction force on the front wheels (N)
*F*_*tr*_: Traction force on the rear wheels (N)
*F*_*zf*_: Vertical force of front tires (N)
*F*_*zr*_: Vertical force of rear tires (N)
*f*_*xf*_: Rolling resistance of front tires (N)
*f*_*xr*_: Rolling resistance of rear tires (N)
*α*_*f*_: Side-slip angles of the front tires (degrees)
*α*_*r*_: Side-slip angles of the rear tires (degrees)
*ω*: Yaw velocity of tractor (degrees s^−^^1^)
*β*: Carbody sideslip angle (degrees)

## Abbreviations

GPS: Global position system
IMU: Inertial measurement unit
RTK: Real-time kinematic
CG: Centre of gravity
